# ER-stress-induced secretion of circulating glucose-regulated protein 78kDa (GRP78) ameliorates pulmonary artery smooth muscle cell remodelling

**DOI:** 10.1007/s12192-022-01292-y

**Published:** 2022-08-27

**Authors:** Muntadher Al Zaidi, Carmen Pizarro, Carolin Bley, Elena Repges, Alexander Sedaghat, Sebastian Zimmer, Felix Jansen, Vedat Tiyerili, Georg Nickenig, Dirk Skowasch, Adem Aksoy

**Affiliations:** Heart Center Bonn, Department of Internal Medicine II, University Hospital Bonn, Venusberg-Campus 1, 53127 Bonn, Germany

**Keywords:** Pulmonary arterial hypertension, Vascular remodelling, ER stress, Chaperone

## Abstract

Pulmonary arterial hypertension (PAH) is driven by vascular remodelling due to inflammation and cellular stress, including endoplasmic reticulum stress (ER stress). The main ER-stress chaperone, glucose-regulated protein 78 kDa (GRP78), is known to have protective effects in inflammatory diseases through extracellular signalling. The aim of this study is to investigate its significance in PAH. Human pulmonary arterial smooth muscle cells (PASMC) were stimulated with compounds that induce ER stress, after which the secretion of GRP78 into the cell medium was analysed by western blot. We found that when ER stress was induced in PASMC, there was also a time-dependent secretion of GRP78. Next, naïve PASMC were treated with conditioned medium (CM) from the ER-stressed donor PASMC. Incubation with CM from ER-stressed PASMC reduced the viability, oxidative stress, and expression of inflammatory and ER-stress markers in target cells. These effects were abrogated when the donor cells were co-treated with Brefeldin A to inhibit active secretion of GRP78. Direct treatment of PASMC with recombinant GRP78 modulated the expression of key inflammatory markers. Additionally, we measured GRP78 plasma levels in 19 PAH patients (Nice Group I) and correlated the levels to risk stratification according to ESC guidelines. Here, elevated plasma levels of GRP78 were associated with a favourable risk stratification. In conclusion, GRP78 is secreted by PASMC under ER stress and exhibits protective effects from the hallmarks of PAH in vitro. Circulating GRP78 may serve as biomarker for risk adjudication of patients with PAH.

**Graphical abstract:**

Proposed mechanism of ER-stress-induced GRP78 secretion by PASMC. Extracellular GRP78 can be measured as a circulating biomarker and is correlated with favourable clinical characteristics. Conditioned medium from ER-stressed PASMC reduces extensive viability, ROS formation, inflammation, and ER stress in target cells. These effects can be abolished by blocking protein secretion in donor cells by using Brefeldin A. 
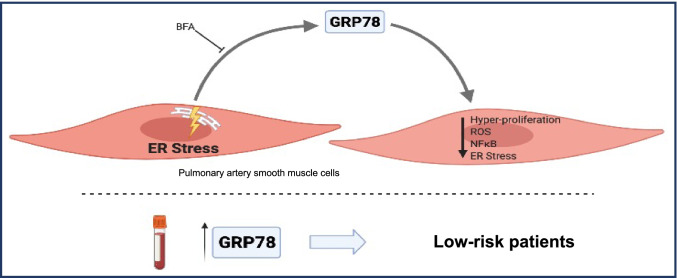

**Supplementary Information:**

The online version contains supplementary material available at 10.1007/s12192-022-01292-y.

## Introduction

Pulmonary arterial hypertension (PAH) is characterized by progressive vascular remodelling of the pulmonary arteries, thus causing an increase in pulmonary arterial pressure and, consequently, right heart failure (Rabinovitch 2012; Thenappan et al. 2018). Excessive proliferation of pulmonary arterial smooth muscle cells (PASMC) and chronic inflammation strongly contribute to the progression of the disease (Huertas et al. 2020). Due to its devastating consequences, there is unmet need for prognosis parameters and novel therapeutic targets for PAH.

The endoplasmic reticulum (ER) is a central organelle that is necessary for the synthesis, folding, and modification of cellular proteins. This crucial task is carefully controlled by physiological conditions and any perturbations to this homeostasis promote ER stress and, subsequently, an accumulation of misfolded proteins. This, in turn, activates a well-orchestrated cellular process known as the unfolded-protein response (UPR). The ER chaperone glucose-regulated protein 78 kDa (GRP78, also known as BiP: binding immunoglobulin protein) serves as the main regulator of the UPR by sensing misfolded proteins and then inducing differential signalling cascades. The aim of the UPR is to restore the cell to physiological conditions, but prolonged activation of the UPR leads to inflammatory and apoptotic signalling. ER stress and excessive UPR activation are involved in the pathogenesis of a large variety of diseases, including cancer, atherosclerosis, and PAH (Zhao and Ackerman 2006; Hotamisligil 2010; Hetz 2012). Animal studies suggest that supplementation of chemical chaperones could be a feasible option for treating PAH (Dromparis et al. 2013; Koyama et al. 2014).

In recent years, novel functions of GRP78 have been proposed. It was shown in one study that ER stress also triggers extracellular secretion of GRP78 (Delpino and Castelli 2002). Biomarker studies have proven that GRP78 is elevated in patients with obesity and metabolic syndrome, both related to chronic inflammation (Girona et al. 2019). Moreover, administration of GRP78 was found to resolve inflammation in mononuclear cells in vitro (Corrigall et al. 2004; Qin et al. 2017). Additionally, GRP78 was found to exhibit disease-modifying and immunomodulatory effects in murine models of rheumatoid arthritis (RA) (Brownlie et al. 2006; Zaiss et al. 2019). These results suggest a pivotal and protective role for extracellular GRP78 on cellular inflammation.

Thus far, data on the secretion and extracellular effects of GRP78 in PAH are lacking. The aim of this study was, first, to analyse the role of GRP78 in in vitro models of PAH and, secondly, to analyse its potential as a biomarker in a cohort of patients suffering from PAH.

## Methods

### Cell culture

Primary human pulmonary artery smooth muscle cells (PASMC) were purchased (PromoCell, Germany; Cat# C-12521) and cultured in their respective cell medium (PromoCell, Cat# C-22062) under standard conditions (37°C, 5% CO_2_, 100% relative humidity). Once the cells reached 70–90% confluence, the medium was removed, and the cells were washed once before passaging by using trypsinisation. The cells used for experiments were from passages 6–8.

Additionally, PASMC were cultured under hypoxic conditions in an incubator (37°C, 1% O_2_, 5% CO_2_, 100% relative humidity) for 24 h. Hypoxia-challenged cells were otherwise treated as described above.

### ER-stress induction and conditioned medium

PASMC (“donor cells”) were seeded into 6-well plates and stimulated with vehicle (DMSO), tunicamycin (5 μg/ml) or thapsigargin (0.1 μM) for 4, 6, 12, 24, or 48 h in serum-containing medium. Additionally, PASMC were stimulated for 48 h with either tunicamycin or thapsigargin combined with Brefeldin A (BFA), an inhibitor of Golgi-ER protein trafficking. The cell medium was aspirated and centrifuged at 800×g for 5 min to remove cellular debris. The supernatant was frozen at -80°C until it was used as conditioned medium. For the cell-culture experiments, new PASMC (“target cells”) were treated with a combination of 60% fresh, serum-containing medium, and 40% of the respective pre-warmed conditioned medium (CM). The optimal ratio of these media was previously determined by viability experiments, which found that the cell viability was unaltered when using the 60:40 ratio. The effects of CM from cells under ER stress on different cell types were previously investigated (Mahadevan et al. 2011; Blackwood et al. 2020).

In some experiments, PASMC were incubated for 48 h with different concentrations of recombinant GRP78 (Prospec Bio, Cat# HSP-037) that was diluted in DMSO. Thereafter, the readout experiments were conducted as described below.

### Protein isolation and western blot

For the analysis of extracellular proteins, the cell medium was collected and incubated for 10 min at 4°C with one volume of 100% trichloroacetic acid per four volumes of protein sample, then centrifuged at 14,000× g for 5 min. The supernatant was removed and the remaining cell pellet was washed twice with 200 μl cold acetone followed by centrifugation at 14,000× g for 5 min. The pellet was then dried for 5 min at 95°C before adding 100μl H_2_O and 30μl 2x Laemmli buffer (4% SDS, 10% ß-mercaptoethanol, 20% glycerol, 0.004% bromophenol blue, 0.125 M Tris HCl pH 6.8) and the sample loaded onto an SDS-PAGE gel (Bio-Rad, CT# 456-1084).

Intracellular proteins from cells in the same well were isolated by lysing with RIPA Buffer (Sigma-Aldrich, Cat# R0278) containing 1:25 Protease Inhibitor Cocktail (Roche, Cat# 4693132001). The lysates were centrifuged at 13,000×g for 10 min at 4°C. The supernatant was removed and the protein concentration was measured by using a Qubit Protein Assay (Thermo Fisher Scientific, Cat# Q33211) in a Qubit-4 Fluorometer (Thermo Fisher Scientific). Twenty micrograms of the resulting protein were loaded onto an SDS-PAGE gel.

Electrophoresis was performed by using a Mini Protean system (Bio-Rad), and the proteins were transferred to a nitrocellulose membrane (Carl Roth, HP40.1) for western blotting. The membrane was blocked with 5% BSA for 1 h at room temperature before it was incubated overnight with primary antibodies at 4°C.

The next day, the membranes were washed three times with 0.1% tris-buffered saline containing 0.1% Tween 20. Then, secondary antibodies that were conjugated with horseradish peroxidase (HRP) were added for 1 h at room temperature. The membrane was then washed again three times before detection was performed with an ECL Western Blot Detection Reagent (Sigma-Aldrich, Cat# RPN2232) on a ChemoCam HR16-3200 Imager (Intas).

The following antibodies were used: GRP78 (dilution 1:1000, Cell Signaling Technology, Cat# 3177S), ß-actin (dilution 1:2000, Sigma-Aldrich, Cat# A1978), anti-mouse-IgG secondary antibody (dilution 1:10,000, Sigma-Aldrich, Cat# A9044), and anti-rabbit-IgG secondary antibody (dilution 1:10,000, Sigma-Aldrich Cat# A0545).

### Viability assay

alamarBlue^TM^ HS Cell Viability Reagent (Thermo Fisher Scientific, USA; Cat# A50100) was added to cells treated by CM or recombinant GRP78. The mixtures of viability reagent and cells were incubated for 4 h under standard culture conditions, as outlined above, and protected from light. Then, the absorbance was measured by using an Infinite M200 Microplate Reader (Tecan, Switzerland). Alamarblue is a resazurin-based solution that is reduced to the fluorescent form resorufin by cells that are undergoing respiration and thus are viable.

### Reactive oxygen species (ROS) measurement

The formation of ROS was measured by using a 2′,7′-dichlorofluorescein diacetate (DCFDA, Sigma-Aldrich, USA; Cat# D6883) assay. For this assay, cells were seeded one day prior to the experiment unto a dark-bottomed 96-well microplate, protected from light, and were treated with CM or recombinant GRP78. After 24 h, the cells were stimulated with 75 μM hydrogen peroxide (H_2_O_2_) for 1 h. Then, DCFDA was added at a final concentration of 50 μM for 45 min. Finally, the supernatant containing DCFDA was removed and the cells were washed before the addition of 100μl PBS. Fluorescence was detected immediately using a microplate reader with a maximum excitation and emission of 492 nm and 527 nm, respectively.

### Quantitative real-time polymerase chain reaction

Total RNA was isolated from the cells by using TRIzol^TM^ (Thermo Fisher Scientific, Cat# 15596018) and chloroform phase separation. The amount of RNA collected was quantified using a Nanodrop spectrophotometer (Nanodrop Technologies, USA). Then, 0.5–2 μg of RNA were reverse transcribed by using an Omniscript RT Kit (Qiagen, Cat# 205113). Finally, quantitative real-time PCR was performed on a 7500 HT Real-Time PCR machine (Applied Biosystems, USA) by using the TaqMan gene expression assays (Thermo Fisher Scientific) and Gene Expression Master Mix (Thermo Fisher Scientific, Cat# 4369542). CT values up to 40 were used for analysis and all samples were run in triplicate. The values were analysed using the ΔΔCT method, by normalising to 18S ribosomal RNA.

The following TaqMan Assays were used: Interleukin-6 (IL-6, Hs00174131_m1), nuclear factor “kappa-light-chain-enhancer” of activated B-cells (NF-κB, Hs00765730_m1), glucose-regulated protein 78 (GRP78, Hs00607129_gH), C/EBP homologous protein (CHOP, Hs00358796_g1), hypoxia inducible factor α (HIFα, Hs00153153_m1), and 18S ribosomal RNA (18S, Hs99999901_s1).

### Subjects

For the prospective study, consecutive PAH patients routinely presenting to our tertiary care university hospital outpatient clinic were enrolled. In all patients, PAH was previously confirmed by using right-heart catheterization, with a mean pulmonary artery pressure (mPAP) ≥ 25 mmHg. Patients were categorized according to the Nice classification (Simonneau et al. 2019) and only patients with Nice Group I were enrolled.

Risk stratification was performed according to Boucly et al., who proposed to use a number of low-risk criteria to facilitate an accurate prediction of prognosis in PAH (Boucly et al. 2017; Hoeper et al. 2018). Therefore, we assessed six different criteria: 6-min walk distance (6MWD), WHO functional class (FC), N-terminal pro-brain natriuretic peptide (NT-proBNP) level, right atrial pressure (RAP), cardiac index (CI), and central venous oxygen saturation (SvO_2_). Using the cut-off values proposed in the current ESC guidelines (Galiè et al. 2016), we defined the low-risk criteria as 6MWD > 440m, WHO FC I or II, NT-proBNP < 300 ng/L, RAP < 8 mmHg, CI ≥ 2.5 L/(min*m^2^), and SvO_2_ > 65%, with the first three criteria being assessed at baseline and also at follow-up. RAP, CI, and SvO_2_ were only reassessed at follow-up if there was a clinical indication for repeated right-heart catheterization.

The study complied with the principles of the declaration of Helsinki and was approved by the local ethics committee. Written informed consent was obtained from all patients.

### Blood sampling and biomarker measurement

Blood was drawn from a peripheral vein of PAH patients routinely presenting to our outpatient clinic and collected directly into EDTA tubes. The samples were kept on ice and centrifuged (1500×g, 15 min at 4°C) for a maximum of 30 min. Thereafter, plasma was transferred into coded aliquots and frozen at −80°C until the time of analysis.

The plasma concentrations of GRP78 (Enzo Life Sciences, Inc, Farmingdale, New York, USA) and HSP27 (R&D Systems, Bio-Techne, Minneapolis, USA) were analysed by using commercially available enzyme-linked immunosorbent assay (ELISA) kits. 4-Parameters Logistic Regression (Graphpad Prism 9.0.0) was used to plot the concentrations.

### Statistical analysis

Continuous variables are presented as means ± the standard deviation if normally distributed and as the median and interquartile range if not normally distributed, as assessed with the Kolmogorov–Smirnov test. Depending on the distribution, continuous variables were tested using either a Student’s *t* test or Mann–Whitney *U* test. Simple linear models (ANOVA) or the Kruskal–Wallis-test were used for more than two groups. Categorical variables are given as absolute numbers and percentages. Differences in categorical variables were assessed by the use of Fisher’s exact test. For correlation analyses, bivariate correlation was assessed by using Pearson’s r.

Statistical analyses were performed with SPSS version 25 (IBM Corporation, Somer, NY) and GraphPad Prism (9.0.0). Statistical significance was considered as a 2-tailed probability value ≤0.05. All authors vouch for the data and analyses.

## Results

### GRP78 is secreted by PASMC under ER stress

**Fig. 1:**
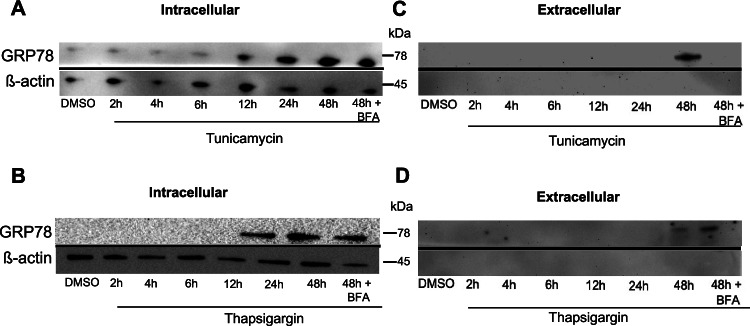
GRP78 is secreted by PASMC. **A** Representative western blot of PASMC treated with DMSO, tunicamycin (2, 4, 6, 12, 24, 48 h, 5 μg/ml) or tunicamycin (48 h, 5 μg/ml) and BFA (5 μg/ml). Left panel: intracellular proteins. **C** Extracellular proteins. Incubated with antibodies against GRP78 or ß-actin (housekeeping intracellular protein). **B** Representative western blot of PASMC treated with DMSO, thapsigargin (2, 4, 6, 12, 24, 48 h, 0.1 μM) or thapsigargin (48 h, 0.1 μM) and BFA (5 μg/ml). Left panel: intracellular proteins. **D** Extracellular proteins. Incubated with antibodies against GRP78 or ß-actin (housekeeping intracelluar protein). *n* = 3

PASCM were treated with the inducers of ER stress tunicamycin (5 μg/ml) or thapsigargin (0.1 μM) for 2, 4, 6, 12, 24, or 48 h (Abdullahi et al. 2017). Treatment with tunicamycin and thapsigargin promoted intracellular GRP78 expression in a time-dependent manner (Figure 1A,B). Interestingly, also when we determined the extracellular concentration of GRP78 in the cell medium, there was a time-dependent increase in its expression (Figure 1C,D). Significant expression of GRP78 could be detected in the medium after 48 h of either tunicamycin or thapsigargin treatment (Figure 1C,D). The housekeeping (and strictly intracellular) protein ß-actin was not detected in the extracellular medium, indicating that the GRP78 detected in the medium is not a result of apoptotic cell debris. Finally, co-treatment with Brefeldin A (BFA), an inhibitor of ER–Golgi protein trafficking, abolished GRP78 expression in the cell medium of tunicamycin (Figure 1C), but not thapsigargin (Figure 1D), treated cells. These results suggest that there is active ER–Golgi-dependent secretion of GRP78 by tunicamycin treated PASMC.

### GRP78 containing CM reduces the viability of PASMC under the stimulation of PDGF-BB

To investigate the effect of secreted GRP78, we established a model utilising conditioned medium (CM) collected from ER-stressed donor PASMC. The target PASMC were incubated with CM from donor PASMC treated with DMSO (control), tunicamycin, tunicamycin + BFA, thapsigargin, or thapsigargin + BFA for 48 h. Utilizing this model, we analysed the effects of a presence or absence of secreted proteins (including GRP78) from ER-stressed donor cells on target cells.

**Fig. 2:**
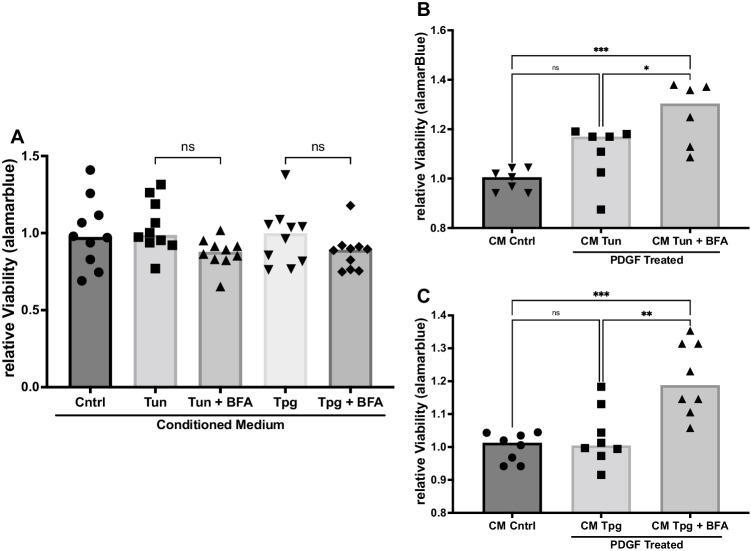
ER-stress conditioned medium modulates viability. **A** Viability of PASMC measured by alamarblue® assay, after treatment with conditioned medium (CM) for 24 h. *n* = 10. **B** Viability of PASMC measured by alamarblue® after treatment with PDGF (30 ng/ml) for 48 h, followed by treatment with CM (control CM, tunicamycin CM: CM Tun, or tunicamycin + Brefeldin A CM: CM Tun + BFA) for 24 h. *n* = 7. **C** Viability of PASMC measured by alamarblue®, after treatment with PDGF (30 ng/ml) for 48 h, followed by treatment with CM (control CM, thapsigargin CM: CM Tpg, or thapsigargin + BFA CM: CM Tpg + BFA) for 24 h. *n* = 8. Simple linear models (ANOVA). *p < 0.05, **p < 0.01, ***p < 0.001, ****p <0.0001

It is known that excessive proliferation and viability of PASMC are major contributor to PAH. We found no difference in the basal viability of the target cells when treated with the above-mentioned CM (Fig. 2A). In the next step, PAH conditions were simulated by incubating cells with 30 ng/ml platelet-derived growth factor BB (PDGF) for 48 h. In the final 24 h of the PDGF treatment, the respective CM was added. PASMC treated with tunicamycin CM had significantly lower viability than PASMC treated with CM of tunicamycin + BFA donor cells (CM Cntrl: 1.0-fold vs. CM Tun 1.10 vs. CM Tun + BFA 1.262, ANOVA *p*=0.0008, Fig. 2B). These effects were mirrored when the cells were treated with CM from thapsigargin/thapsigargin + BFA-treated donor cells (CM Cntrl: 1.0-fold vs. CM Tpg 1.03 vs. CM Tpg + BFA 1.21, ANOVA *p*=0.0001, Fig. 2C).

In another approach, target PASMC were treated with the respective CM under hypoxic conditions (1% O_2_, 5% CO_2_ for 24 h) to mimic the conditions of PAH. We could not detect a difference in viability under these conditions (Supplementary Fig. S1A, B).

### ER-stress conditioned medium modulates the formation of reactive oxygen species (ROS)

**Fig. 3:**
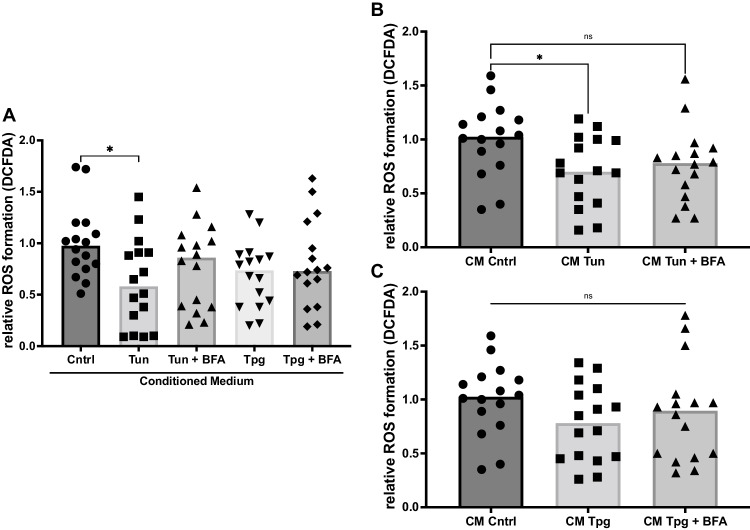
Reactive Oxygen Species (ROS) formation is altered by conditioned medium from cells under ER stress. **A** ROS formation in PASMC, measured by DCFDA assay, after treatment with conditioned medium (CM) for 24 h. *n* = 16. **B**, **C** ROS formation in PASMC, measured by DCFDA assay, after treatment with CM for 24 h and simultaneous treatment with 75 μM H_2_O_2_ for 1 h. *n* = 16. Simple linear models (ANOVA). *p < 0.05, **p < 0.01, ***p < 0.001, ****p <0.0001

ROS formation has repeatedly been demonstrated to be involved in the development of PAH (Sommer et al. 2016). Hence, we measured ROS formation, by using DCFDA assays, in PASMC that were treated with the CM as described above. Under basal conditions (Figure 3A) and when mimicking oxidative stress by treating the target with H_2_O_2_ (Figure 3B,C), PASMC treated with tunicamycin CM, but not tunicamycin + BFA CM, had significantly lower ROS formation than the control CM (Figure 3A: CM Cntrl: 1.0-fold vs. CM Tun 0.61 vs. CM Tun + BFA 0.78, ANOVA *p*=0.029; Fig. 3B: CM Cntrl: 1.0-fold vs. CM Tun 0.71 vs. CM Tun + BFA 0.76, ANOVA *p*=0.039). There was no difference in ROS formation between thapsigargin and thapsigargin + BFA CM under both conditions (Fig. 3A,C).

### GRP78 containing CM alters inflammatory gene expression in target cells

**Fig. 4:**
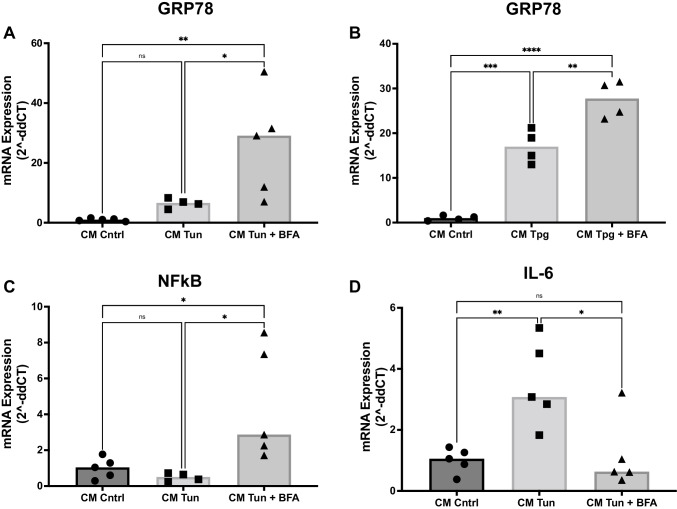
Conditioned medium from cells under ER stress regulates gene expression in target cells. **A**, **B** mRNA expression levels of GRP78 in target PASMC, treated for 24 h with conditioned medium (CM) from tunicamycin- (**A**) or thapsigargin- (**B**) stimulated donor PASMC. *n* = 3–5. **C**, **D** mRNA expression levels of NF-κB in target PASMC, treated for 24 h with conditioned medium (CM) from tunicamycin- (**C**) or thapsigargin- (**D**) stimulated donor PASMC. *n* = 3-5. *n* = 4–5. **E**, **F** mRNA expression levels of IL-6 in target PASMC, treated for 24 h with conditioned medium (CM) from tunicamycin- (**E**) or thapsigargin- (**F**) stimulated donor PASMC. *n* = 3-5. *n* = 5. mRNA expression was measured by qPCR and quantified by using the 2^-ddCT^ method. Simple linear models (ANOVA). *p < 0.05, **p < 0.01, ***p < 0.001, ****p <0.0001

Next, we sought to elucidate the effects of CM from cells under ER stress on mRNA expression in target cells. CM from donor cells that were additionally treated with BFA significantly increased GRP78 expression in the target cells compared to tunicamycin/thapsigargin alone (Fig. 4A: CM Cntrl: 1.0-fold vs. CM Tun 6.5-fold vs. CM Tun + BFA 26.0-fold; Fig. 4B: CM Cntrl: 1.0-fold vs. CM Tpg 17.1-fold vs. CM Tun + BFA 27.7-fold). There was no significant difference in the expression of CHOP mRNA, another marker of ER stress (Supplementary Fig. S2A, B).

NF-κB is a transcription factor involved in moderating cellular inflammation. Activation of NF-κB is known to occur during the development of PAH (Raychaudhuri et al. 1999). NF-κB expression was unaltered between treatments with CM from control cells or tunicamycin treated cells, but administration of CM from tunicamycin + BFA-treated PASMC increased NF-κB expression in target cells (Fig. 4C: CM Cntrl: 1.0-fold vs. CM Tun 0.49-fold vs. CM Tun + BFA 4.55-fold, ANOVA *p*=0.016).

Another inflammatory marker involved in PAH pathogenesis is interleukin-6 (IL-6). Interestingly, CM from tunicamycin treated PASMC increased IL-6 mRNA expression compared to control CM or tunicamycin + BFA CM (Fig. 4E: CM Cntrl: 1.0-fold vs. CM Tun 3.5-fold vs. CM Tun + BFA 1.2-fold, ANOVA *p*=0.005). There was no significant difference detectable regarding NF-κB and IL-6 expression between thapsigargin CM and thapsigargin + BFA CM treated cells (Figure S2C, S2D).

### Recombinant GRP78 mimics the effect of CM on gene expression

**Fig. 5:**
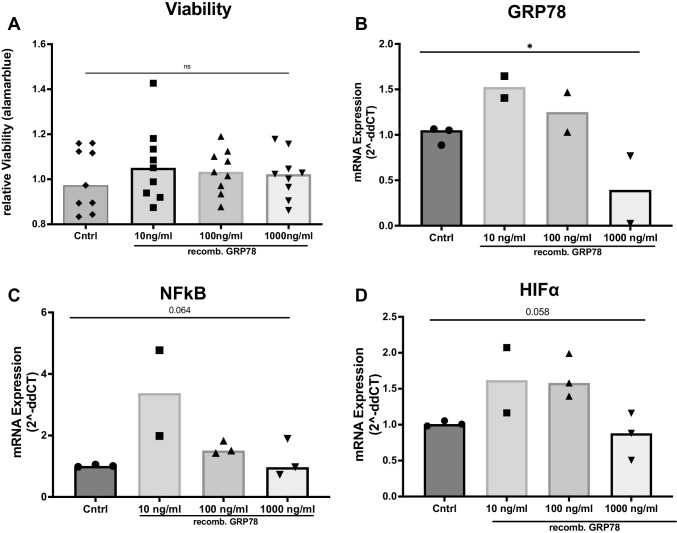
Effects of recombinant GRP78 on viability and mRNA expression. **A** Viability of PASMC measured by alamarblue® assay after treatment with recombinant GRP78 (10 ng/ml, 100 ng/ml, 1000 ng/ml for 48 h). *n* = 9. **B**, **C**, **D** mRNA expression of GRP78 (**A**), NF-κB (**B**), and HIFα (**C**) in target PASMC, treated with recombinant GRP78 (10 ng/ml, 100 ng/ml, 1000 ng/ml for 48 h). *n* = 3. mRNA expression was measured by qPCR and quantified by using the 2^-ddCT^ method. Simple linear models (ANOVA). *p < 0.05, **p < 0.01, ***p < 0.001, ****p <0.0001

Next, we explored if direct treatment with recombinant GRP78 can exhibit similar effects on PASMC as treatment with CM. Therefore, PASMC were incubated with recombinant GRP78 (48 h, 10 ng/ml, 100 ng/ml, or 1000 ng/ml). There was no difference in viability under basal conditions (Figure 5A: Cntrl: 1.0-fold vs. 10 ng/ml GRP78: 1.06-fold vs. 100 ng/ml GRP78: 1.04-fold vs. 1000 ng/ml GRP78: 1.02-fold, ANOVA *p*=0.36) and also no difference in viability after PDGF stimulation or hypoxia treatment (Supplementary Fig. S3A and B). By analysing gene expression, we found that adding recombinant GRP78 decreased the expression of GRP78 mRNA in target cells in a dose-dependent manner (Figure 5B: Cntrl: 1.0-fold vs. 10 ng/ml GRP78: 1.52-fold vs. 100 ng/ml GRP78: 1.25-fold vs. 1000 ng/ml GRP78: 0.39-fold, ANOVA *p*=0.048). There was also a similar alteration in NF-κB and HIFα expression (Figure 5C,D). Intriguingly, the lower concentrations of recombinant GRP78 caused an increase in expression of these markers and higher concentrations of GRP78 reduced expression, which shows a trend toward the dose-dependancy of this effect.

### Circulating GRP78 in PAH patients

Nineteen consecutive patients with PAH corresponding to Nice group I were included into the study. Baseline characteristics of the PAH patients are presented in Table 1 (female 52%, mean age of 62.5 ± 17 years). The majority of patients were classified as WHO FC II (42.1%) or III to IV (47.3%) and were predominantly receiving PAH combination therapy (68.4%). The mean pulmonary artery pressure (mPAP) was 37.29 ± 13.06 mmHg, with a pulmonary arterial wedge pressure (PAWP) of 11.2 ± 3.5 mmHg and a pulmonary vascular resistance (PVR) of 6.1 ± 3.1 Wood units. At the time of enrollment into our study, 68.4% (13/19) of patients had more than two low-risk risk factors (“low-risk” patients) and 31.6% (6/19) presented with zero to two low-risk factors (“high-risk” patients). Low-risk patients had lower PVR (4.8 ± 2.7 vs. 8.8 ± 1.6, *p* = 0.001) and were more likely to have COPD or asthma as a comorbidity.

**Table 1:** Baseline characteristics

	All patients (*n*=19)	Low risk (*n*=13)	High risk (*n*=6)	*p*-value
Age, years	62.5 ± 17	59.2 ± 19	69.8 ± 7	0.10
Female sex	10 (52.6%)	8 (61.5%)	2 (33.3%)	0.25
WHO FC				
I	2 (10.5%)	2 (15.4%)	0	0.41
II	8 (42.1%)	6 (46.2%)	2 (33.3%)	
III to IV	9 (47.3%)	5 (26.3%)	4 (66.6%)	
6MWD, m	404 ± 131	423 ± 151	363 ± 65	0.25
NT-proBNP, ng/ml	818 ± 1361	636 ± 1394	1211 ± 1317	0.41
Comorbidities				
Arterial hypertension	11 (57.9%)	8 (61.5%)	3 (50%)	0.64
Diabetes	4 (21.1%)	2 (15.4%)	2 (33.3%)	0.77
CKD	4 (21.4%)	3 (23.1%)	1 (16.7%)	0.75
Atrial fibrillation	5 (26.3%)	3 (23.1%)	2 (33.3%)	0.47
COPD/asthma	8 (42.1%)	8 (61.5%)	0	**0.01**
Previous MI	1 (5.3%)	0	1 (16.7%)	0.13
Haemodynamics				
RAP, mmHg	10.0 ± 5.6	7.8 ± 5.1	10.2 ± 3.1	0.25
SvO_2,_ %	66.5 ± 8.7	67.6 ± 7.5	64.0 ± 6.9	0.41
CI, L/(min×m^2^)	2.65 ± 0.72	2.83 ± 0.70	2.24 ± 0.62	0.09
mPAP, mmHg	37.9 ± 13.6	34.1 ± 13.0	46.2 ± 11.8	0.07
PAWP, mmHg	11.2 ± 3.5	11.6 ± 3.1	10.2 ± 4.3	0.49
PVR, Wood units	6.1 ± 3.1	4.8 ± 2.7	8.8 ± 1.6	**0.001**
PAH therapy				
ERA	15 (78.9%)	11 (84.6%)	4 (66.6%)	0.37
PDE-5i/sGC	15 (78.9%)	9 (69.2%)	6 (100%)	0.13
PCA	5 (26.3%)	2 (15.4%)	3 (50%)	0.11
Monotherapy	6 (31.6%)	5 (38.5%)	1 (16.7%)	0.36
Combination therapy	13 (68.4%)	8 (61.5%)	5 (83.3%)	0.34
GRP78	1172 ± 374	1346 ± 299	796 ± 202	**0.0008**

Values are *n* (%) or mean ± SD. *WHO FC*, World Health Organization Functional Class; *6MWD*, 6-min walking distance; *NT*-*proBNP*, N-terminal pro-B-type natriuretic peptide; *CKD*, chronic kidney disease; *COPD*, chronic obstructive pulmonary disease; *MI*, myocardial infarction; *RAP*, right atrial pressure; *SvO*_*2*_, central venous oxygen saturation; *CI*, cardiac index; *mPAP*, mean pulmonary arterial pressure; *PAWP*, pulmonary arterial wedge pressure; *PVR*, pulmonary vascular resistance; *ERA*, endothelin receptor antagonist; *PDE-5i*, phosphodiesterase type 5 inhibitor; *sGC*, soluble guanylyl cyclase stimulator; *PCA*, prostacyclin analogue. *p*-value determined by Student’s *t* test or chi-square test.

**Fig. 6:**
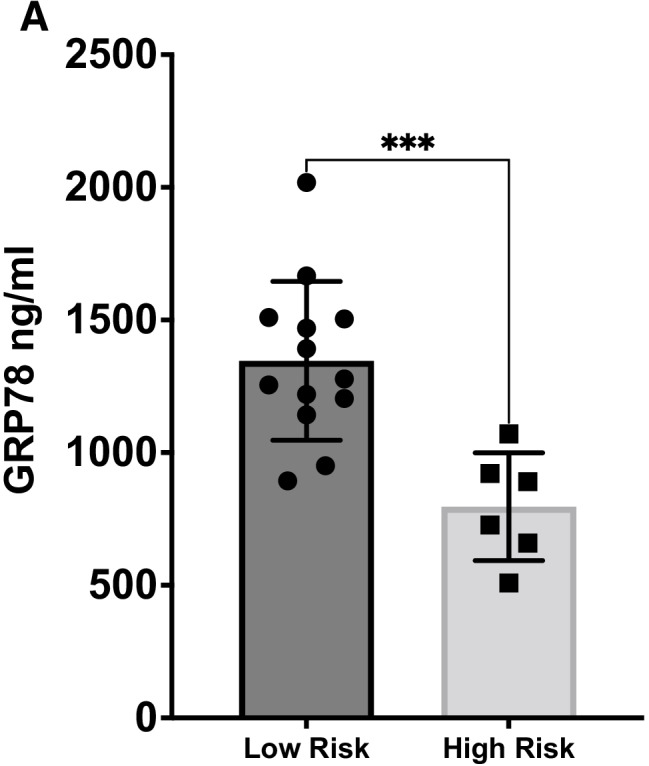
GRP78 as a biomarker in PAH patients. **A** GRP78 concentration in PAH patients of Nice Group I with a low number of low-risk criteria (0–2, “high-risk” patients) and a higher number of low-risk criteria (3–6, “low-risk” patients). *n* = 19. Student’s *t* test. *p < 0.05, **p < 0.01, ***p < 0.001, ****p <0.0001

Interestingly, low-risk patients had significantly higher GRP78 plasma levels (1346 ± 299 ng/ml vs. 796 ± 203 ng/ml, *p* = 0.0008, Fig. 6A). However, there was no association between low-risk criteria and plasma levels of heat shock protein 27 (HSP27), another prominent chaperone with well-known extracellular properties in cardiovascular disease (Supplementary Fig. 4A). There was no difference in GRP78 levels when comparing patients with PAH monotherapy and combination therapy (Monotherapy: 1199 ± 346 ng/ml, combination therapy: 1160 ± 399.8 ng/ml, *p* = 0.833).

We furthermore measured GRP78 plasma levels in ten healthy volunteers with a mean age of 21.9 ± 2.3 years, 40% were male. GRP78 plasma concentration was below the assay detection limit of 25 ng/ml in all volunteers. Next, we mined available biostatistic data of plasma proteomics in humans (Farrah et al. 2011). Most of the used data sets were gathered from healthy individuals. The normal GRP78 plasma concentration was estimated to be 100 ng/ml.

## Discussion

Because of its devastating effects on patients, finding novel therapeutic targets for PAH is highly important. Here, we have demonstrated that PASMC actively secrete GRP78 in response to the induction of ER stress. GRP78-containing conditioned medium from ER-stressed PASMC dampens viability, oxidative stress, NF-κB activation, and ER stress in target cells. Intercellular transmission of ER stress signals, e.g. by connexins, has been well-characterized and is mostly attributed deleterious properties (Tirosh et al. 2021). However, thus far, there is only scarce evidence examining any potential protective effects of the intercellular responses to ER stress. Our patient-derived data shows that circulating levels of the ER-stress protein GRP78 strongly correlate with favourable risk stratification in PAH. Our study introduces a novel, anti-inflammatory feedback mechanism that occurs during pulmonary vascular ER stress.

We found that incubating cells with the well-established ER-stress inducers tunicamycin and thapsigargin promoted the secretion of GRP78. Utilising a similar approach, Blackwood et al. found that thapsigargin, but not tunicamycin, promoted GRP78 secretion in cardiomyocytes (Blackwood et al. 2020). In our study, both ER-stress inducers promoted extracellular GRP78 secretion. Interestingly, however, we found that treatment with BFA inhibited tunicamycin-, but not thapsigargin-induced secretion. These results indicate that there are cell-type specific effects on ER-stress-induced protein secretion. In the current study, administration of both tunicamycin and thapsigargin in donor cells modulated the viability and GRP78 expression of target cells, while the effects on ROS formation and expression of inflammatory markers were only observed when using tunicamycin CM. Tunicamycin induces ER stress by directly inhibiting N-linked glycosylation of proteins while thapsigargin depletes the Ca^2+^ levels in the ER by inhibiting the endoplasmic reticulum Ca^2+^ ATPase. Thus, it appears that the mechanism of ER stress induction in donor cells influences the effects exhibited in target cells. These findings might be explained by specific secretion of additional, yet unknown, proteins. Additionally, thapsigargin-induced secretion of GRP78 is not altered by inhibition of the canonical ER–Golgi protein trafficking, suggesting the existence of non-canonical protein secretion pathways. For example, secretion by exosomes is another well-known way of protein secretion. Exosomes can be secreted by donor cells by membrane budding and contain proteins, lipids, and nucleic acids that deliver signals to recipient cells (Pegtel and Gould 2019). Secretion of GRP78-containing extracellular vesicles was previously described to play a role in vascular biology (Furmanik et al. 2021).

In addition, Blackwood et al. found GRP78 secretion to exert paracrine, cytoprotective effects on cardiomyocytes (Blackwood et al. 2020). Since myocardial dysfunction of the right ventricle is an important issue in clinically advanced PAH (Olsson et al. 2018), these proposed cardioprotective properties are of great clinical significance. Our data supports the idea that GRP78 secretion serves as a protective feedback mechanism, induced by cardiovascular ER stress.

Under basal conditions, we found no alteration of cell viability in PASMC treated with CM containing or not containing GRP78. But when the cells were stimulated with the PAH-promoting growth factor PDGF (Schermuly et al. 2005), an increase in viability was more pronounced in PASMC treated with CM where GRP78 secretion was inhibited. Direct treatment with recombinant GRP78 did not alter the viability under basal or PAH-promoting conditions. Thus, GRP78 alone does not limit the viability of cells and only limits PASMC viability in combination with disease-promoting growth factors. These findings are important to be able to exclude toxicity to cells, such as coronary smooth muscle cells. Moreover, additional components of the conditioned medium are required to exert anti-proliferative effects on target cells. Intracellular GRP78 exists in a multi-protein complex and might also be secreted in a similar complex (Meunier et al. 2002). Hence, modulation of the secretion of GRP78 rather than direct GRP78 treatment might be a more feasible therapeutic option. In a previously published study using hypoxia-challenged rats, treatment with the peptide intermedin increased cellular GRP78 levels and attenuated PASMC proliferation and PAH development (Mao et al. 2014). This study further supports our findings.

When analysing gene expression, the absence of GRP78 in the CM promoted mRNA expression of GRP78 in target cells. Hence, the protective properties of extracellular GRP78 might be mediated by a reduction of the intracellular activation of UPR in target cells. However, there were no alterations in CHOP expression, hinting at a modulation of distinct UPR pathways. Vascular inflammation is a well-established feature of PAH and NF-κB was frequently ascribed a prominent role in initiating pulmonary vascular inflammation (Fiordelisi et al. 2019; Huertas et al. 2020). Intriguingly, we found that an absence of GRP78 in the CM promotes NF-κB expression in target cells. When analysing IL-6 expression, we found that the presence of GRP78 in CM increased IL-6 mRNA. There are conflicting reports regarding the role of IL-6 in PAH biology. However, a recent multicentre analysis found circulating IL-6 to be secreted by PASMC and to be associated with positive clinical outcomes (Simpson et al. 2020).

To the best of our knowledge, this is the first study measuring circulating ER-stress proteins in patients with PAH. We found that elevated GRP78 levels are associated with favourable risk stratification. These findings are highly relevant because a dramatic, rapid deterioration is frequent in patients with PAH (Galiè et al. 2016). For risk stratification, we used the number of low-risk criteria present to stratify patients. This approach was found to be more accurate than an approach using the average risk category for prognosis prediction in PAH patients (Hoeper et al. 2018).

Contrarily, there was no association between HSP27, another chaperone with well-established extracellular signalling properties in CVD (Seibert et al. 2013), and risk stratification. These results emphasize a specific, presumably protective, role for GRP78 in PAH. Previous studies have reported alterations of plasma GRP78 levels in a variety of diseases (Girona et al. 2019; Doerflinger et al. 2020). In a recent study from our group, we found GRP78 to have beneficial prognostic implications in patients undergoing transcatheter aortic valve replacement (Aksoy et al. 2021). However, there is so far only scarce knowledge regarding the biological significance of elevated plasma levels.

Limitations of our study are the limited number of patient samples and the lack of animal data, e.g. of PAH model animals. Additionally, in vitro PAH conditions do not a hundred percent resemble in vivo the vascular environment in PAH in humans. Furthermore, we only analysed PASMC and did not additionally examine endothelial cells. Moreover, in this current study we mainly focussed on the effects of GRP78-containing CM and did not perform in-depth analysis of the mechanisms of GRP78 secretion. While we found that Tunicamycin-induced secretion is most likely mediated by canonical ER-Golgi transport, the mechanism of Thapsigargin-induced secretion remains unknown. Future studies have to focus on additional ways of secretion including exosomes and usage of specific GRP78-antibodies.

Our results are in line with other studies proposing an anti-inflammatory effect for extracellular GRP78 (Shields et al. 2011; Zaiss et al. 2019). In human trials, treatment with GRP78 was well-tolerated and did not induce toxicity in rheumatoid arthritis patients (Kirkham et al. 2016). Because of its presumably protective effects on both cardiomyocytes and PASMC, GRP78 administration may be a feasible therapeutic option in patients with PAH. An additional therapeutic approach might be direct modulation of GRP78 secretion. Future studies are required to further characterize the mechanisms of secretion and action of GRP78. Moreover, larger clinical cohorts are needed to elucidate the significance of circulating GRP78 as a biomarker for PAH.

## Supplementary Information

ESM 1 (PDF 207 kb)

## Data Availability

The data that support the findings of this study are available from the corresponding author upon reasonable request.
