# Supplementary figures and images for: ER-stress-induced secretion of circulating glucose-regulated protein 78kDa (GRP78) ameliorates pulmonary artery smooth muscle cell remodelling
Source: Cell Stress Chaperones. 2022 Aug 27;27(5):561–72. doi: 10.1007/s12192-022-01292-y (PMC9485380; doi:10.1007/s12192-022-01292-y)

## **Supplementary Figures**

Figure S1

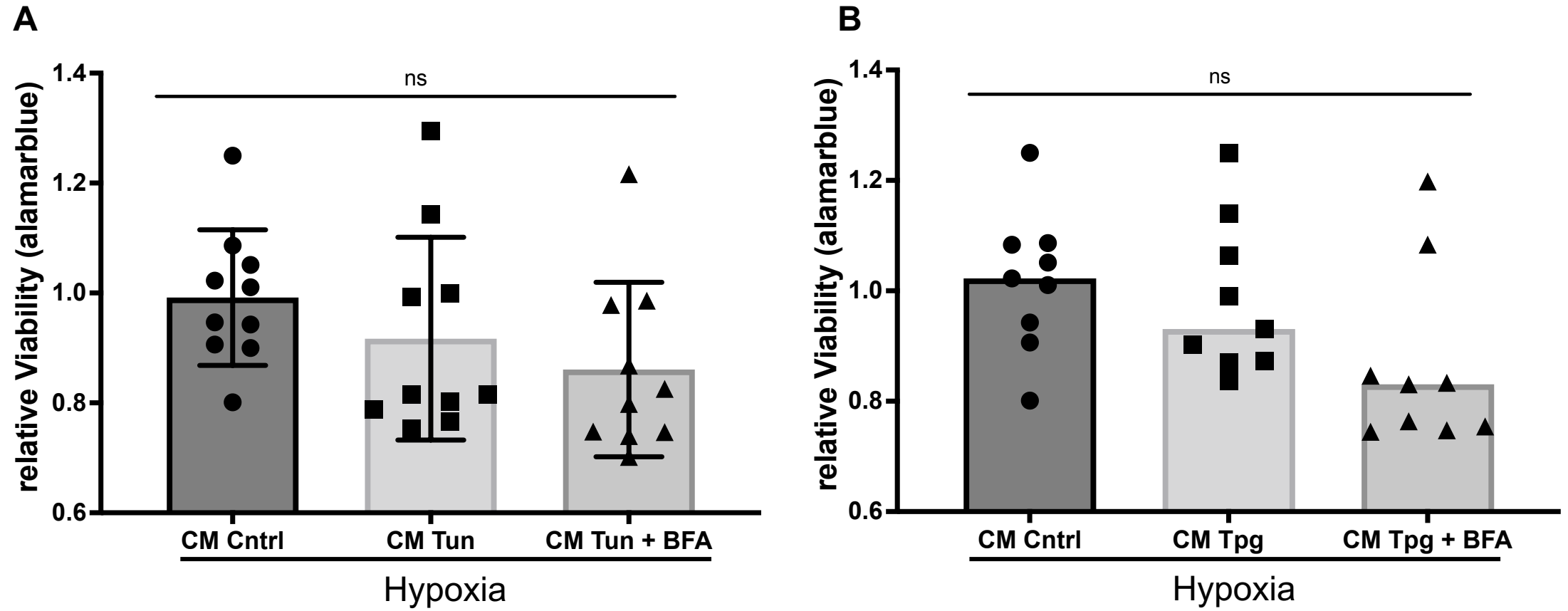

Figure S2

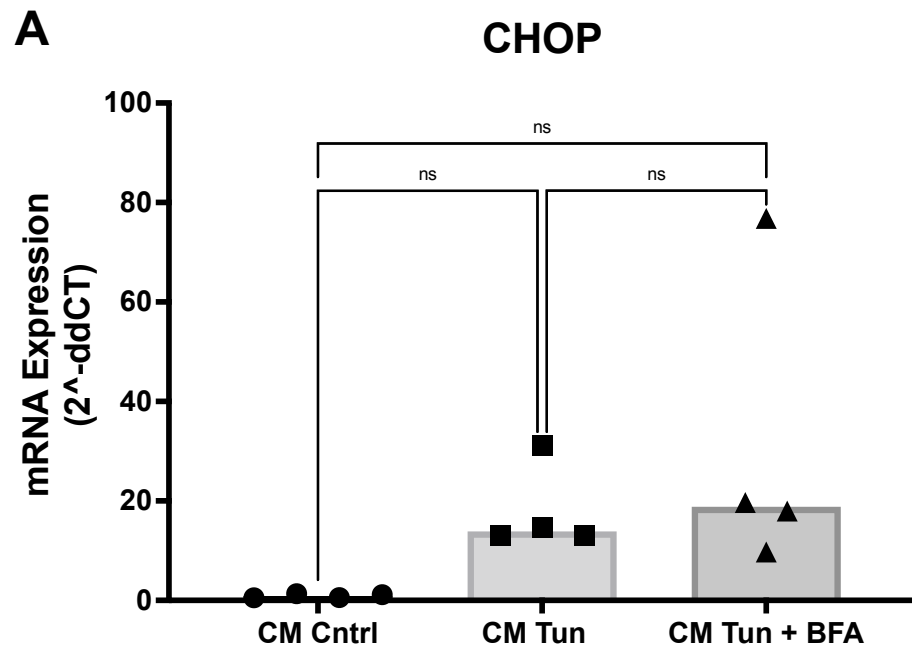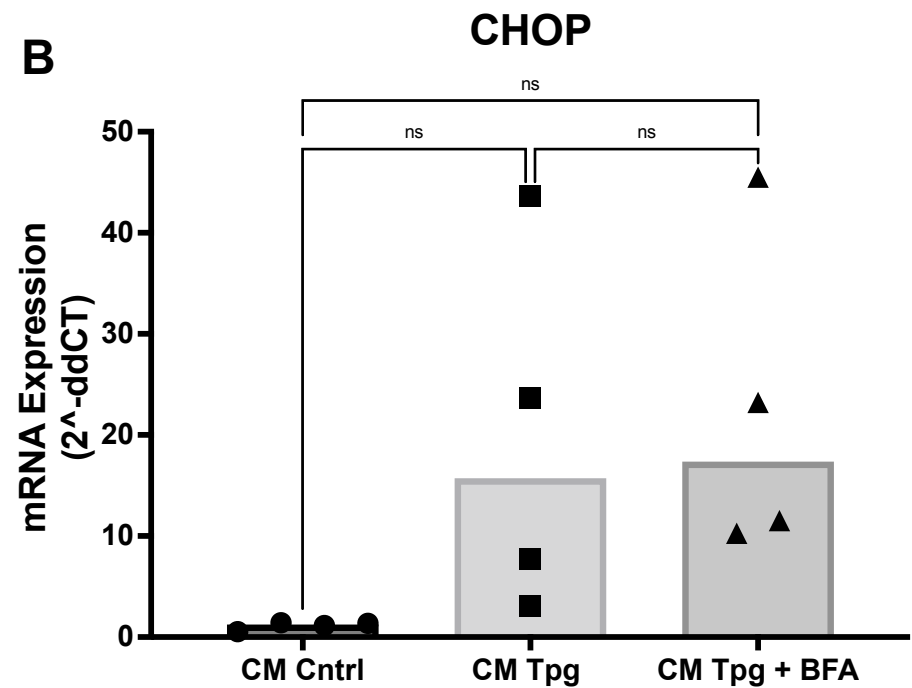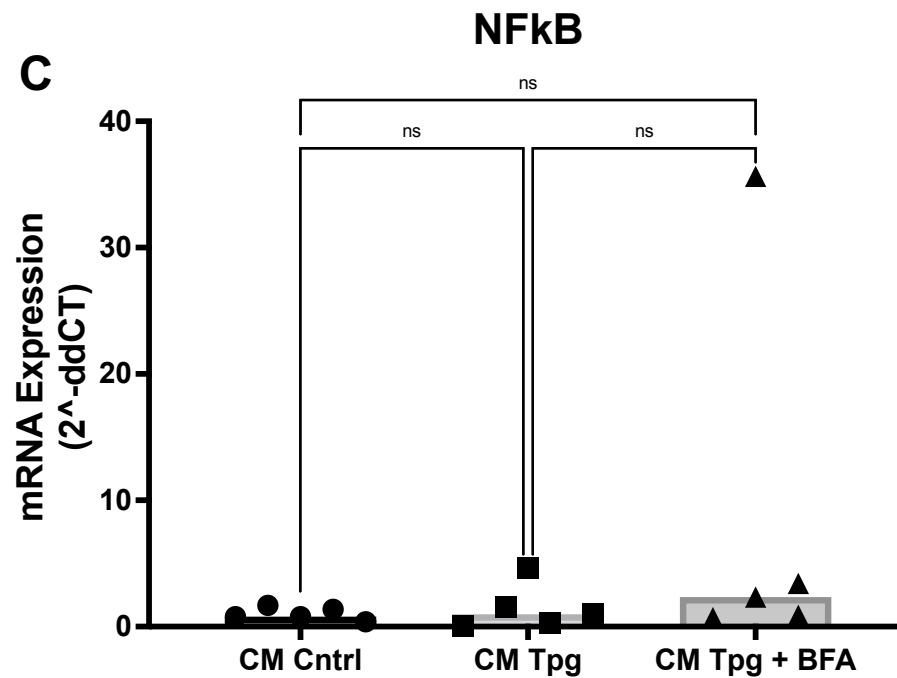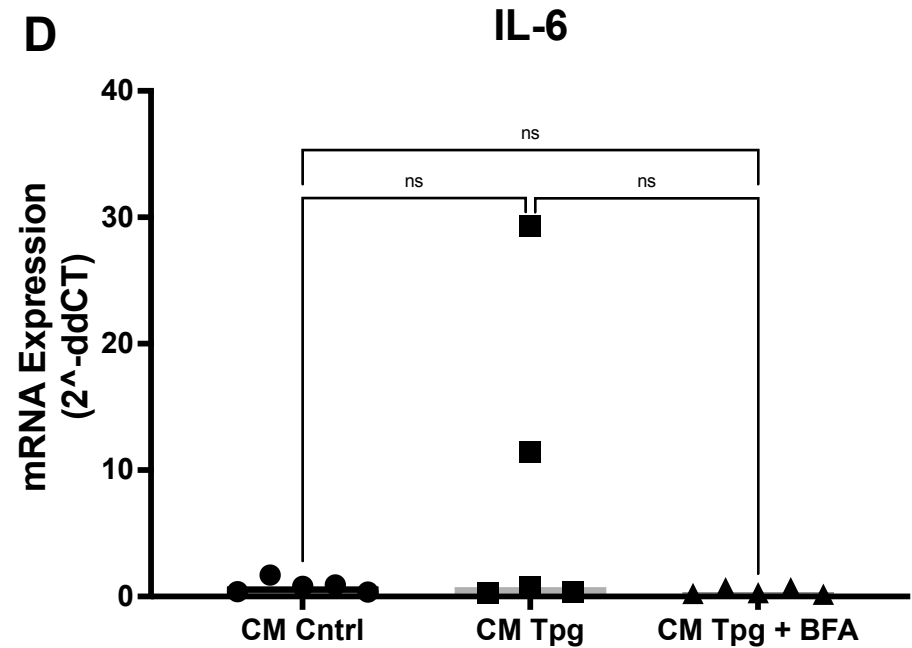

Figure S3

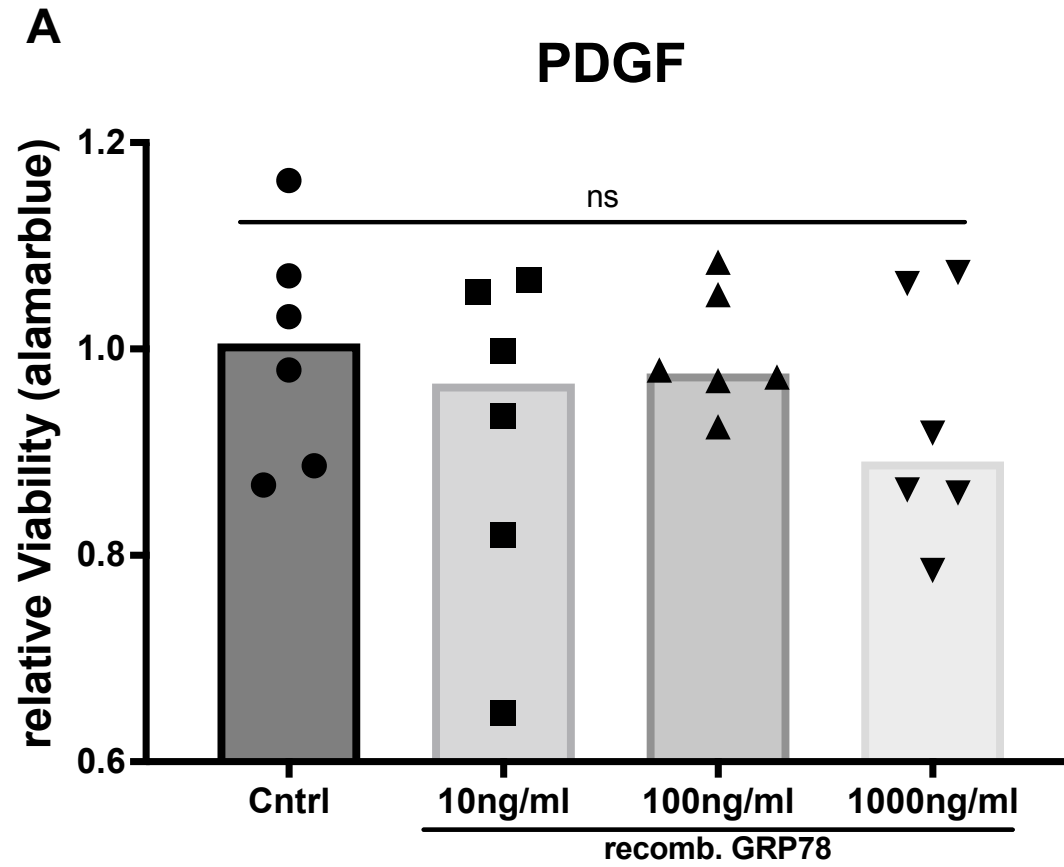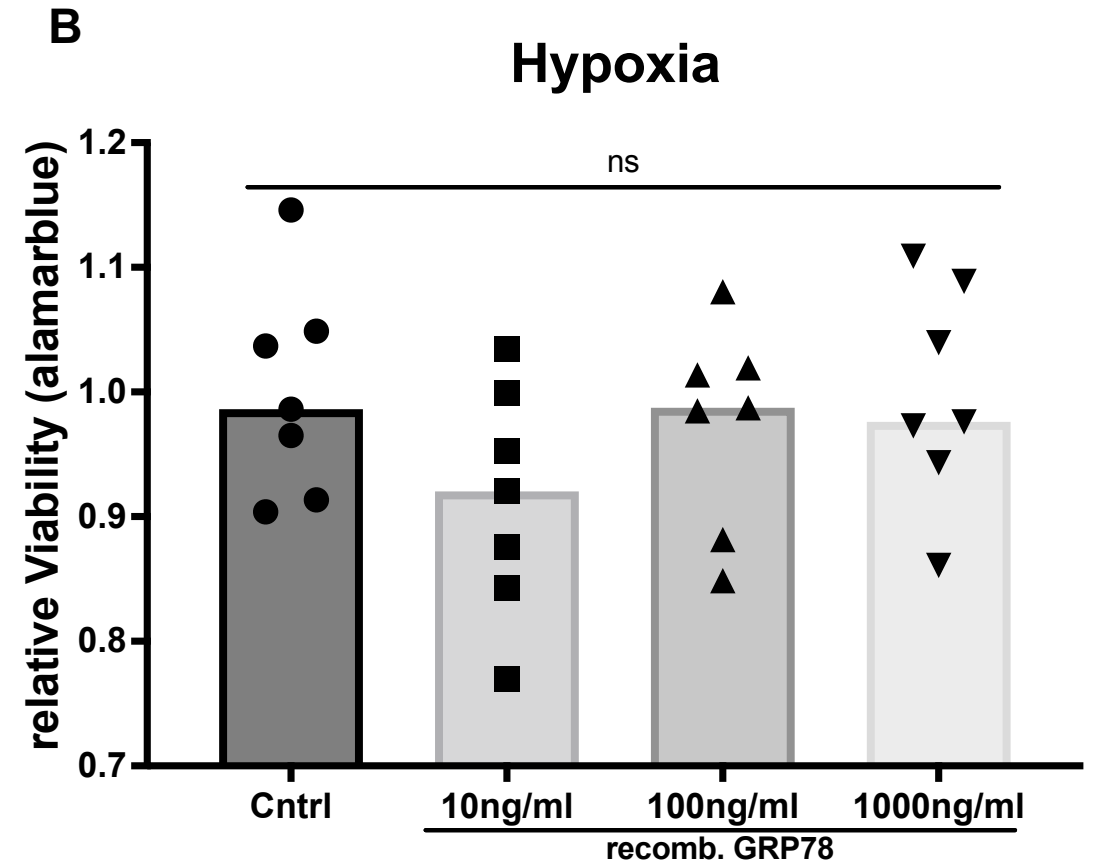

Figure S4

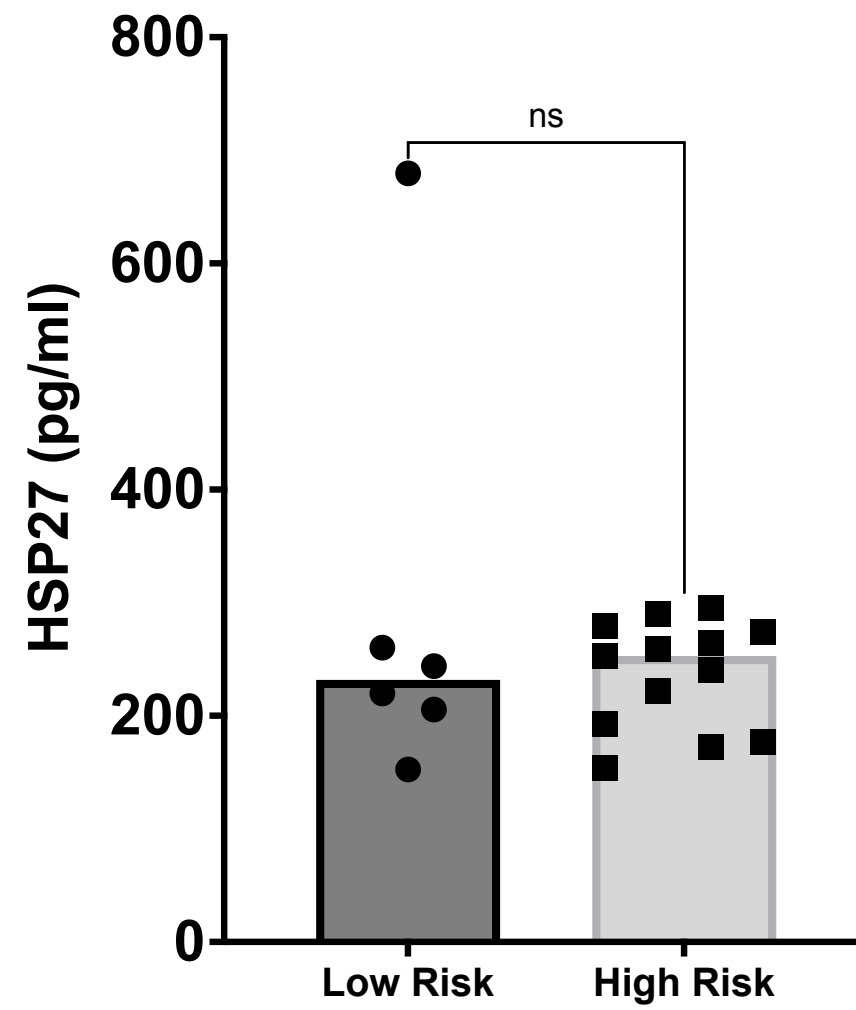

Supplement: Supplementary file 1 — (PDF 207 kb) [file 12192_2022_1292_MOESM1_ESM.pdf]
